# Electroacupuncture at ST36-ST37 and at Ear Ameliorates Hippocampal Mossy Fiber Sprouting in Kainic Acid-Induced Epileptic Seizure Rats

**DOI:** 10.1155/2014/756019

**Published:** 2014-06-22

**Authors:** Chung-Hsiang Liu, Yi-Wen Lin, Hsin-Cheng Hsu, Hsu-Jan Liu, Wan-Jung Lin, Ching-Liang Hsieh

**Affiliations:** ^1^Graduate Institute of Integrated Medicine, College of Chinese Medicine, China Medical University, 91 Hsueh-Shih Road, Taichung 40402, Taiwan; ^2^Department of Neurology, China Medical University Hospital, Taichung 40402, Taiwan; ^3^Research Center for Chinese Medicine & Acupuncture, China Medical University, Taichung 40402, Taiwan; ^4^Graduate Institute of Acupuncture Science, College of Chinese Medicine, China Medical University, Taichung 40402, Taiwan; ^5^Department of Chinese Medicine, China Medical University Hospital, Taichung 40402, Taiwan; ^6^Graduate Institute of Chinese Medicine, College of Chinese Medicine, China Medical University, Taichung 40402, Taiwan

## Abstract

Our previous study showed that mossy fiber sprouting can occur in the hippocampus region in rats 6 wk after kainic acid-induced epileptic seizure, and this mossy fiber sprouting can facilitate epileptogenesis. Transcutaneous auricular vagal nerve stimulation (VNS), which is similar to cervical VNS, can reduce the occurrence of epileptic seizure in intractable epilepsy patients. Greater parasympathetic nerve activity can be caused by 2 Hz electroacupuncture (EA). Therefore, we investigated the effect of 2 Hz EA at ST-36-ST37 and at the ear on mossy fiber sprouting in kainic-treated Sprague-Dawley rats. The results indicated that applying 2 Hz EA at ST36-ST37 and at the ear for 3 d per week over 6 consecutive weeks can ameliorate mossy fiber sprouting in the hippocampus region of rats. These results indicated that applying 2 Hz EA at ST36-ST37 and at the ear might be beneficial for the treatment and prevention of epilepsy in humans.

## 1. Introduction

Our previous study showed that mossy fiber sprouting can be observed in the dentate gyrus of the hippocampus in rats at 6 wk after intraperitoneal injection of kainic acid (KA) [1]. Proper et al. (2000) showed that the hippocampal sclerosis develops in patients with temporal lobe epilepsy and that the hippocampal neuron loss associated with mossy fiber sprouting might contribute to epileptogenesis [2]. The mossy fiber sprouting score of the dentate gyros correlates positively with the frequency of seizure recurrence in pilocarpine-induced seizure and status epilepticus mice [3], indicating that mossy fiber sprouting increases the network excitability in the hippocampus and dentate gyrus. The mossy fiber spouting could be the result of epileptic discharge, which causes synaptic reorganization [4]. Collectively, the formation of mossy fiber sprouting in the hippocampus following a seizure is related to epileptogenesis (i.e., spontaneous seizure).

Cervical vagus nerve stimulation (VNS) can reduce the frequency of seizure in patients with drug-resistant epilepsy [5], and the effect of VNS can occur because it can restore the functional balance or synchronization of the pathological area [6]. Auricular sensory afferent includes the vagus, glossopharyngeal, and facial nerves, which exhibit parasympathetic nerve activity that is similar to that of cervical VNS [7]. Transcutaneous auricular VNS can reduce the occurrence of seizure in pediatric patients with intractable epilepsy [8], and auricular acupuncture can cause parasympathetic tone [9]. Acupuncture at* Shaofu* (HT8) can reduce hippocampal neuronal death and inhibit inflammatory reaction in KA-treated mice [10]. Our previous study showed that applying 2 Hz electroacupuncture (EA) stimulation to the bilateral* Zusanli* (ST36) and* Shangjuxu* (ST37) points can reduce the pulse rate, indicating that this application might enhance parasympathetic activity [11].

Therefore, this study investigated how applying 2 Hz EA at ST36-ST37 and at the ear affects mossy fiber sprouting and hippocampal neuronal cells in KA-induced epileptic rats.

## 2. Material and Methods

### 2.1. Animal Models

Adult male Sprague-Dawley (SD) rats weighing 200–300 g (Biol-Asco Taiwan Co. Ltd., Taiwan) were housed in a 12 h light-dark cycle room in the Animal Center of China Medical University. The room's ambient temperature (25°C ± 1°C) was controlled by an air conditioner. The rats were allowed to move freely, and they were supplied with adequate food and water. The experimental procedures were conducted in accordance with the* Guideline Principles for the Care and Use of Laboratory Animals*.

### 2.2. Establishing the Epileptic Animal Model

#### 2.2.1. Electrode Preparation

Electrodes were prepared 4 d prior to the electroencephalogram (EEG) and electromyogram (EMG) recordings, and the procedures were conducted in accordance with our previous research [1]. The rats were intraperitoneally anesthetized with chloral hydrate (400 mg/kg), and the heads of the rats were subsequently fixed in a stereotactic apparatus. An incision was made along the midline of the rat head to expose the scalp. Two stainless screws recording electrodes were placed, respectively, on the left and right sensorimotor cortices, proximal to the epidural. Another electrode was implanted in the frontal sinus service as a reference for the EEG recordings. For the EMG recordings, we inserted bipolar electrode wires through the subcutaneous tissue surrounding the neck muscle. Finally, the electrodes were connected to a relay and subsequently linked to an EEG and EMG recording machine (MP100WSW, BIOPAC System, Inc., Goleta, CA, USA).

#### 2.2.2. The Selection of the Rat and Grouping

Epileptic seizure was induced by intraperitoneally injecting the rat with kainic acid (KA) (12 mg/kg), and seizure was confirmed through behavioral observation and EEG and EMG recordings that were captured using a video recording epileptic behavioral analysis system (SeizureScan, Clever Sys., Inc., Virginia, USA) from 15 min prior to and 3 h after KA administration. The rats that exhibited at least 250 wet dog shakes within 3 h were selected for this study except for normal group without KA injection.

Thirty SD rats were randomly divided into the following 5 groups (6 rats/group). (1) Normal group (NG): the NG rats were intraperitoneally injected with phosphate-buffered saline (PBS) solution (1.0 mL/kg) only. The behavioral seizures and EEG and EMG were recorded 24 h at 7th day in the 5th and 6th wk after KA administration, and then the rats were sacrificed. (2) Control group (CG): the CG rats underwent the same procedure as those in the NG, except that KA (12 mg/kg; Sigma, USA) was used instead of PBS. (3)* Zusanli* group (ZG): the ZG rats underwent the same procedure as those in the CG. In addition, 2 stainless acupuncture needles were inserted into the muscle layer of the bilateral* Zusanli* (ST36) and the* Shangjuxu* (ST37) points (which are similar to those of a human), which were subsequently connected to an EA machine (Trio 300, Japan). To perform the electrical stimulation, a cathode and anode were placed at ST36 and ST37, respectively. The EA (2 Hz in frequency, 150 *μ*s in width, and modulation in mode) was applied, and intensity of stimulus caused the muscle to twitch. The electrical stimulation was delivered 3 times per week over 6 wk continuously under isoflurane anesthesia. Each session lasted for 20 min. (4) Ear group (EG): the procedure for the EG was identical to that of the ZG, except that EA was applied for 10 min to each ear by clip electrodes (the cathode and anode were placed at the apex and lobe, resp.). (5) Sham group (SG): the SG underwent the same procedure as the ZG, except that the needles were inserted subcutaneously, and no electrical discharge was delivered.

#### 2.2.3. Timm's Stain

Timm's staining was performed in accordance with our previous research [1]. First, the brain tissue was prepared. The rats were perfused transcardially (0.37% sodium sulfide solution, 4% paraformaldehyde) while being under chloral hydrate (400 mg i.p.) anesthesia. The brain tissues were postfixed in 4% paraformaldehyde, and the tissues were then immersed in a 30% sucrose solution 2 d prior to sectioning. The brain tissues were removed, then embedded in tissue-freezing medium (OCT) at –80°C, and stored at –20°C in a refrigerator. The brain tissues were cryostat-sectioned, 40 *μ*m coronal sections were used for Timm's staining, and 16 *μ*m coronal sections were used for the immunohistochemical staining (IHC).

The 40 *μ*m sections were stained by immersing them in the developing solution. Subsequently, 1 mL of 17% silver nitrate was added to the solution immediately before the development process. The sections were stained and then washed under running water. Next, the sections were dehydrated using a graded series of ethanol solutions and xylene for 15 min and 5 min, respectively. Finally, the section was coverslipped.

The mossy fiber sprouting in the supragranular molecular layer of the dentate gyrus was quantified based on the staining intensity. Stained mossy fiber sprouting was observed under a Zeiss microscope and the images were captured using a Nikon digital camera. The staining intensity of the mossy fiber sprouting was defined in units of optical density (OD) and analyzed using image analysis software (Image-Pro Plus, Media Cybernetics, Inc., Silver Spring, MD, USA). The OD values were obtained along 2 parallel lines of the inner and outer edges of the inner molecular layer (IML). The margin between the inner and outer edge of the IML of each section was defined as follows:

(1) $$\begin{array}{c} \frac{{\text{OD}}_{o}-{\text{OD}}_{i}}{\left[\left({\text{OD}}_{o}+{\text{OD}}_{i}\right)/2\right]}, \end{array}$$

where OD_*o*_ is the OD value of the outer edge of the IML (i.e., the background value) and OD_*i*_ is the OD value of the inner edge of the IML (i.e., the sprouting mossy fiber terminals). The greater the margin between these 2 values, the higher the density of sprouting mossy fibers.

#### 2.2.4. NeuN IHC Staining

The NeuN IHC staining was performed in accordance with our previous research [1]. First, the sections were washed twice (5 min each) with 0.1 M TRIS buffer (pH, 7.6) and with 1% H_2_O_2_ made in 0.1 M TRIS buffer for 30 min. Subsequently, the sections were washed with 0.1 M TRIS buffer (pH 7.6; 5 min), as well as TRIS A (0.1% Triton X-100 dissolved in 0.1 M TRIS buffer) for 10 min and TRIS B (0.1% Triton X-100 and 0.005% bovine serum albumin [BSA] in 0.1 M TRIS buffer) for 10 min. The sections were blocked in normal goat serum for 45 min and subsequently washed with TRIS A and then with TRIS B (10 min each). The sections were incubated with antiserum (NeuN 1 : 1000, diluted in TRIS B; Chemicon, USA) overnight at 4°C. In the following day, the sections were washed with TRIS A (10 min) and then washed again with TRIS B (10 min). Subsequently, they were incubated with biotinylated goat anti-rabbit immunoglobulin (IG) G (45 min), washed with TRIS A (10 min), and then washed again with TRIS D (0.1% Triton X-100 and 0.005% BSA in 0.5 M TRIS buffer; 10 min). Next, they were incubated with avidin-biotin horseradish peroxidase complex (1 h), washed 3 times with 0.1 M TRIS buffer (5 min each), and then developed in diaminobenzidine tetrahydrochloride (DAB; 1-2 min) before being washed 3 times with 0.1 M TRIS buffer (5 min each). Finally, the sections were incubated with 0.1 M TRIS buffer to stop the reaction. All sections were counterstained with hematoxylin solution, washed 3 times with 0.1 M TRIS buffer, dried, and coverslipped. NeuN-positive staining cells were counted under a light microscope.

#### 2.2.5. GFAP IHC Staining

The GFAP IHC staining was performed in accordance with our previous research [1]. The sections were washed briefly in PBS, treated with 3% H_2_O_2_ in methanol for 30 min at room temperature, blocked with normal animal serum (1 h), rinsed twice with PBS (3 min each), and then incubated with antisera (GFAP 1 : 200 diluted in PBS for 1 h at 37°C; CALBIOCHEM, Germany). The sections were washed 3 times in PBS (3 min each) and then incubated with biotinylated secondary antibody (1 h, room temperature). Subsequently, they were washed 3 times with PBS and incubated with avidin-biotin horseradish peroxidase complex (1 h). They were washed once more in PBS (3 min each), developed in DAB, and then washed in running water to stop the reaction. Finally, the sections were counterstained with hematoxylin solution, washed in running water, dried, and coverslipped. The GFAP-positive stained cells were counted under a light microscope.

#### 2.2.6. Statistical Analysis

The data are presented as mean ± standard deviation (SD), and a one-way analysis of variance with Scheffé's post hoc test was used to examine the differences between the groups. A *P* value less than 0.05 was considered statistically significant.

## 3. Results

### 3.1. KA-Induced Epileptic Seizures in SD Rats

**Figure 1:**
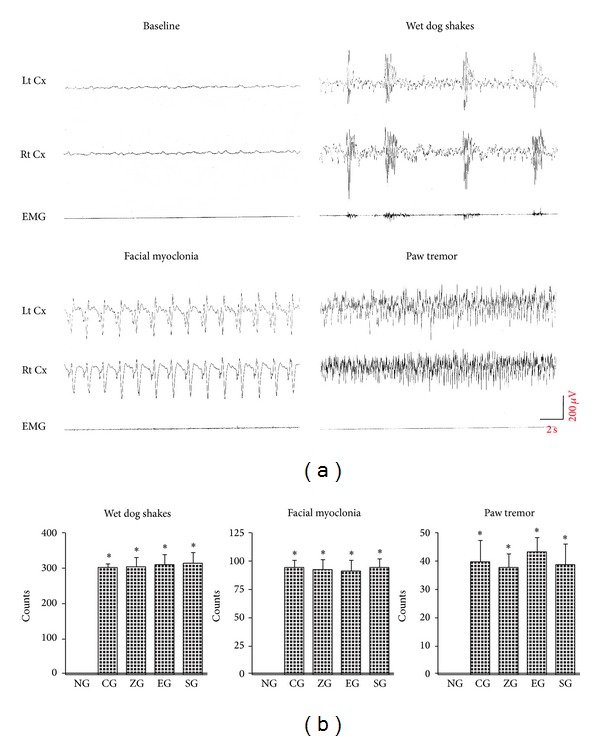
Kainic acid- (KA-) induced epileptic behavior and EEG changes. KA i.p. induced main epileptic behavior, including wet dog shakes, multiple spike-like EEG artifacts, FM with continuous sharp waves, and PT with continuous spikes (a); the count of wet dog shakes, FM, and PT was similar among the CG, ZG, EG, and SG (b). Baseline: baseline EEG activity; NG: normal group; CG: control group; ZG:* Zusanli* group; EG: ear group; SG: sham group.

KA i.p induced mainly epileptic seizure, including wet dog shakes (WDS), facial myoclonia (FM), and paw tremor (PT), and these behaviors exhibited characteristic EEG results (Figure 1(a)). The WDS, FM, and PT counts were greater in the CG, ZG, EG, and SG than in the NG (all *P* < 0.001; Figure 1(b)), whereas the counts between the 2 groups in the CG, ZG, EA, and SG were similar (all *P* > 0.05; Figure 1(b)).

No epileptic seizures were recorded in the 6 rats in the NG for 24 h recordings at 7th day in the 5th and 6th wk after KA administration, whereas 2 rats in the CG, 1 rat in the ZG, 2 rats in the EG, and 1 rat in the SG were found spontaneous seizure.

### 3.2. Effect of EA on Mossy Fiber Sprouting in KA-Induced Epileptic Seizures Rats

**Figure 2:**
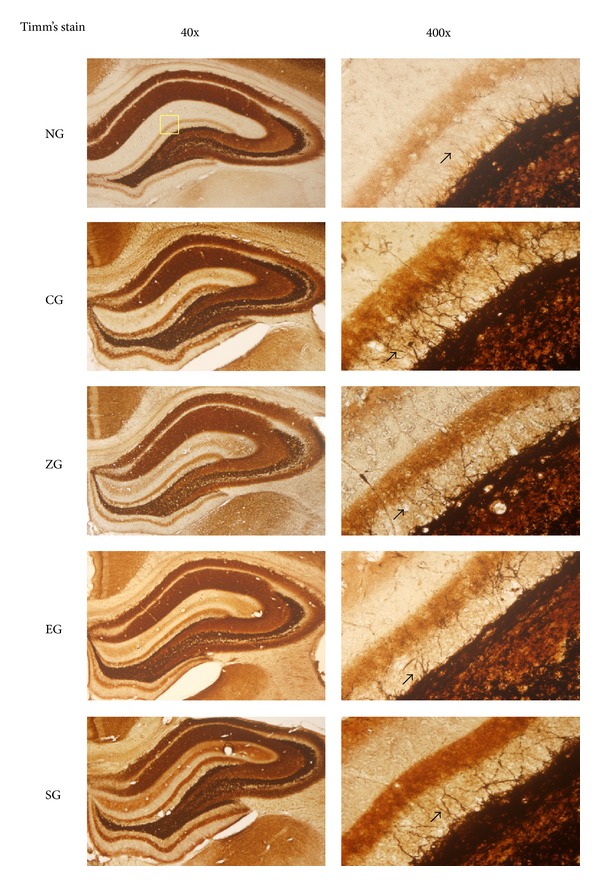
Effect of EA on mossy fiber sprouting in KA-induced epileptic seizure rats. The mossy fiber sprouting (arrowhead) of the hippocampus increased more in the CG than in the NG, and this increase was reduced in the ZG, EA, and SG. NG: normal group; CG: control group; ZG:* Zusanli* group; EG: ear group; SG: sham group.

**Figure 3:**
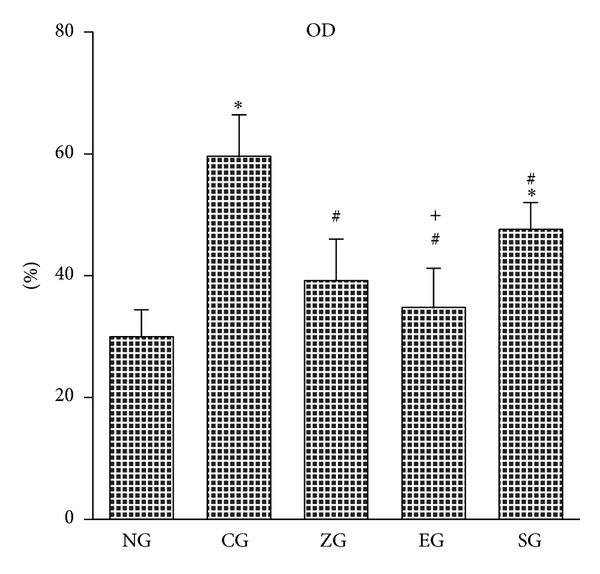
Effect of EA on the optical density (OD) value of mossy fiber sprouting in KA-induced epileptic seizure rats. The OD value of mossy fiber sprouting of the hippocampus increased more in the CG than in the NG, and this increase was reduced in the ZG, EG, and SG. NG: normal group; CG: control group; ZG:* Zusanli* group; EG: ear group; SG: sham group. *P < 0.001 compared with the NG; ^#^ *P* < 0.001 compared with the CG; ^+^ *P* < 0.001 compared with the SG.

The OD value of the mossy fiber sprouting in the CG, EG, and SG was greater than that of the NG (all *P* < 0.001; Figures 2 and 3). The OD value of the mossy fiber sprouting in the ZG, EG, and SG was less than that of the CG (all *P* < 0.001; Figures 2 and 3). The OD value of the mossy fiber sprouting in the EG was less than that of the SG (*P* < 0.001; Figures 2 and 3), whereas the OD value of mossy fiber sprouting was similar between the ZG and SG (*P* > 0.05; Figures 2 and 3).

### 3.3. Effect of EA on the NeuN-Positive Cells in KA-Induced Epileptic Seizures Rats

**Figure 4:**
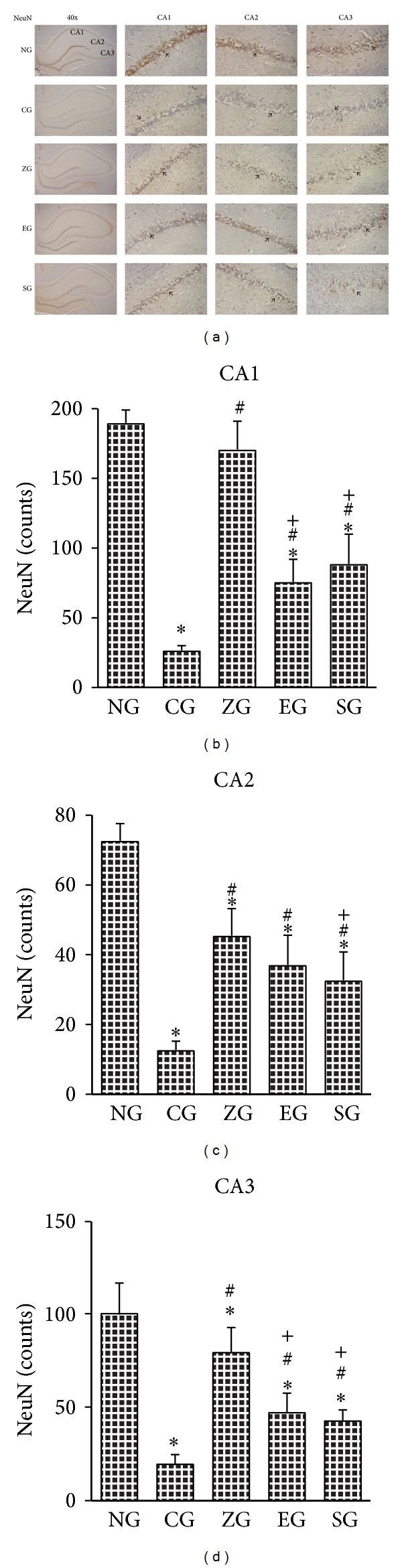
Effect of EA on NeuN-positive cells of the hippocampus in KA-induced epileptic seizure rats. The NeuN-positive cells (arrowhead) of the hippocampus decreased more in the CG than in the NG. This decrease was increased in the ZG, EG, and SG (a). CA1 region of the hippocampus (b); CA2 region of the hippocampus (c); CA3 region of the hippocampus (d). NG: normal group; CG: control group; ZG:* Zusanli* group; EG: ear group; SG: sham group. *P < 0.001 compared with the NG; ^#^ *P* < 0.001 compared with the CG; ^+^ *P* < 0.001 compared with the SG.

In the CA1 area of the hippocampus, the NeuN-positive cell count of the CG was lower than that of the NG, ZG, EG, and SG (all *P* < 0.001; Figures 4(a) and 4(b)). The NeuN-positive cell counts were greater in the NG and ZG than those in the EG and SG (all *P* < 0.001; Figures 4(a) and 4(b)), whereas the NeuN-positive cell counts of the NG and ZG were similar, as were EG and SG (both *P* > 0.05; Figures 4(a) and 4(b)).

In the CA2 area of the hippocampus, the NeuN-positive cell counts of the CG were less than those of the NG, ZG, EG, and SG (all *P* < 0.001; Figures 4(a) and 4(c)). The NeuN-positive cell count was greater in the NG than that of the ZG, EG, and SG (all *P* < 0.001; Figures 4(a) and 4(c)). The NeuN-positive cells were greater in the ZG than those in the SG (*P* < 0.001; Figures 4(a) and 4(c)).

In the CA3 of hippocampus, the NeuN-positive cell count was greater in the NG, ZG, EG, and SG than that of the CG (all *P* < 0.001; Figures 4(a) and 4(d)). The NeuN-positive cell count was greater in the NG than in the ZG, EA, and SG (all *P* < 0.001; Figures 4(a) and 4(d)). The NeuN-positive cell count was greater in the ZG than in the EG and SG (both *P* < 0.001; Figures 4(a) and 4(d)). The NeuN-positive cell counts between the EG and SG were similar (*P* > 0.05; Figures 4(a) and 4(d)).

### 3.4. Effect of EA on the GFAP-Positive Cells in KA-Induced Epileptic Seizures Rats

**Figure 5:**
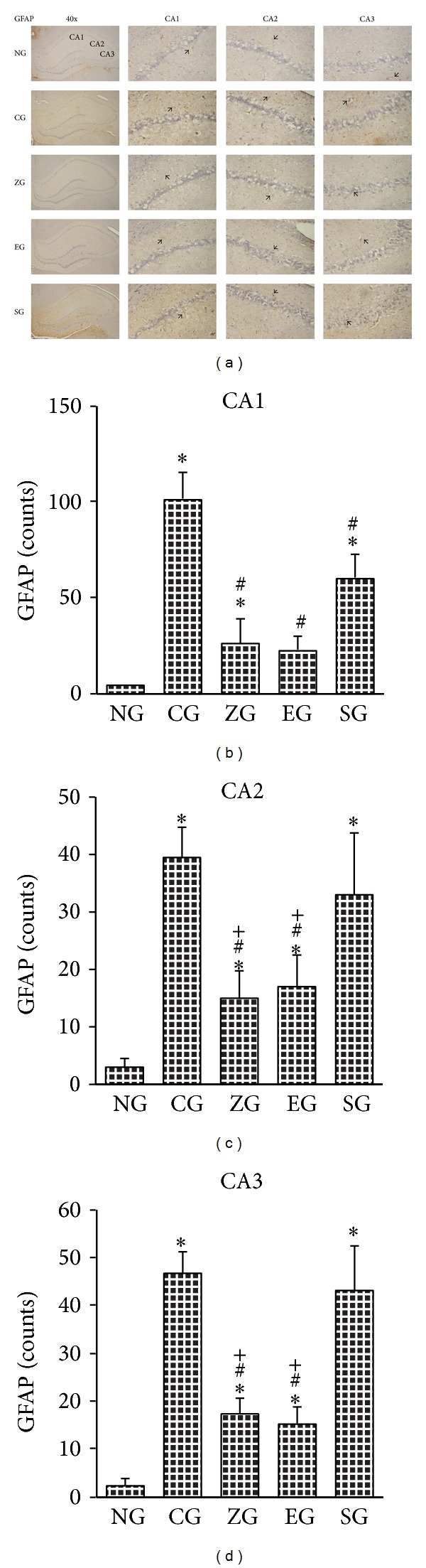
Effect of EA on GFAP-positive cells of the hippocampus in KA-induced epileptic seizure rats. The GFAP-positive cells (arrowhead) of the hippocampus increased more in the CG than in the NG. This increase was reduced in the ZG, EG, and SG (a). CA1 region of the hippocampus (b); CA2 region of the hippocampus (c); CA3 region of the hippocampus (d). NG: normal group; CG: control group; ZG:* Zusanli* group; EG: ear group; SG: sham group. *P < 0.001 compared with the NG; ^#^ *P* < 0.001 compared with the CG; ^+^ *P* < 0.001 compared with the SG.

In the CA1 area of the hippocampus, the GFAP-positive cell count was greater in the CG than in the NG, ZG, EG, and SG (all *P* < 0.001; Figures 5(a) and 5(b)). The GFAP-positive cell was greater in the SG than in the NG, ZG, and EG (all *P* < 0.001; Figures 5(a) and 5(b)). The GFAP-positive cell count was greater in the ZG than in the NG (*P* < 0.001; Figures 5(a) and 5(b)).

In the CA2 of hippocampus, the GFAP-positive cell count was greater in the CG and SG than in the NG, ZG, and EG (all *P* < 0.001; Figures 5(a) and 5(c)). The GFAP-positive cell count was greater in the ZG and EG than in the NG (both *P* < 0.001; Figures 5(a) and 5(c)).

In the CA3 of hippocampus, the GFAP-positive cell count was greater in the CG and SG than in the NG, ZG, and EG (all *P* < 0.001; Figures 5(a) and 5(d)). The GFAP-positive cell was greater in the ZG and EG than in the NG (both *P* < 0.001; Figures 5(a) and 5(d)).

## 4. Discussion

The results of this study indicated that the OD values of the mossy fiber sprouting were greater in the CG than in the NG, ZG, EG, and SG. The OD values of the mossy fiber sprouting were also greater in the SG than in the NG and EG at 6 wk after administering the KA. In addition, the WDS, FM, and PT counts were similar between 2 groups in the CG, ZG, EG, and SG prior to the EA treatment, indicating that the baseline was identical among these groups. Therefore, the results can explain that 2 Hz EA at both the ST36-ST37 and the ear can reduce the formation of mossy fiber sprouting. The role of mossy fiber sprouting has been reported as critical to epileptogenesis, primarily because hippocampal neuronal loss, astrogliosis, and mossy fiber sprouting are more severe in the pharmacoresistant temporal lobe of epilepsy patients with hippocampal sclerosis than in those without hippocampal sclerosis [2]. Abnormal fiber sprouting enhances synaptic circulatory changes, which can promote the generation of spontaneous seizure in a neuronal network model [12]. A positive correlation exists between seizure frequency and the degree of mossy fiber sprouting in transgenic mice based on a pilocarpine-treated model [13]. Therefore, the findings indicate that applying 2 Hz EA at the ST36-ST37 and at the ear can ameliorate epileptogenesis, which is beneficial for the treatment and prevention of epileptic seizures in KA-treated rats.

The mechanism of the vagus nerve stimulation in reducing epileptic seizures is inferred because the vagus nerve is afferent to and terminates in the solitary tract nucleus. Subsequently, the relay information projects into a higher brain region, including the limbic or cortical region, thereby interfering with the EEG synchronization [6, 9] and possibly inducing a cholinergic anti-inflammatory pathway to reduce the inflammatory response [9]. The electrical stimulation of a solitary tract nucleus can increase the gamma aminobutyric acid or reduce the glutamate transmission to suppress epileptic activity [8]. Therefore, we assert that applying 2 Hz EA at the ear can ameliorate mossy fiber sprouting of the hippocampus via multiple pathways and that the auricular-vagal afferent pathway plays a critical role. EA at ST36 can reduce the occurrence of epileptic seizure and supragranular mossy fiber sprouting in a lithium-pilocarpine-induced epileptic seizure rat model [14]; moreover, EA was thought to inhibit seizure via a hypothalamic arcuate nucleus and then through the brain stem to the hippocampus. We hypothesized that ST36-ST37 locate to the anterior tibial muscle could be innervated by a deep peroneal nerve, and the needles were inserted into the subcutaneous region in the SG and innervated by the superficial peroneal nerve. Therefore, both of the acupuncture signals enter the central nervous system via a common peroneal nerve, resulting in the similar effect that was observed between the ZG and SG on mossy fiber sprouting.

Despite our results, the following questions require further explanation: (1) the OD value of the mossy fiber sprouting was similar between ZG and SG, but it was lower in the EG than in the SG; (2) the NeuN-positive cell count was greater in the ZG than in the EG and SG, although the EG and SG were similar; and (3) the GFAP-positive cell count was greater in the CG and SG than in the ZG and EG, whereas the GFAP-positive cell counts of the CG and SG were similar, as were those of the ZG and EG. Therefore, applying 2 Hz EA at the ST36-ST37 or at the ear might produce various effects in KA-treated rats. Applying 2 Hz EA at the ear might be more effective in ameliorating mossy fiber sprouting than 2 Hz EA at ST-36-ST37. Conversely, applying 2 Hz EA at ST36-ST-37 generates a greater effect on neuroprotection than the same treatment applied at the ear. These results agree with those reported by Kang et al. (2013), who showed that the effect of EA on reducing epileptic seizure depends on the stimulus parameters and acupoints [15]. NeuN-positive cells represent neuronal cells [16]. GFAP-positive cells are a marker of astrocyte, and the functional changes of astrocyte play a critical role in epileptogenesis [17–19]. Collectively, this study shows that applying 2 Hz EA both at the ST36-ST37 and at the ear can reduce mossy fiber sprouting and astrocytes and increase neuronal cells in the hippocampus in KA-induced epileptic seizure rats; thus, it is beneficial for the treatment and prevention of epilepsy.

The limitation in this study is the recordings of the behavioral seizures, and EEG and EMG only at 7th day in the 5th and 6th wk after KA administration, and that cannot complete observe the total counts of epileptic seizure around 6 wk after KA administration. Therefore, although 2 rats in the CG, 1 rat in the ZG, 2 rats in the EG, and 1 rat in the SG develop spontaneous seizure is recorded, the real antiepileptic effect of EA remains undetermined. Therefore, complete recordings are needed in the future study.

In conclusion, applying 2 Hz EA at either the ST36-ST37 or the ear could contribute to suppressing the development of epileptogenesis in KA-induced epileptic seizure rats, indicating that this might be suitable for the treatment and prevention of epilepsy in humans.
